# Dosimetric impact of two multileaf collimator systems in simultaneous integrated boost volumetric modulated arc therapy for lymph node metastases in stage IIIC cervical cancer

**DOI:** 10.3389/fonc.2026.1805020

**Published:** 2026-04-23

**Authors:** Hui Xiao, Ang Qu, Haitao Sun, Xile Zhang, Nan Zhang, Shuhua Wei, Xiuwen Deng, Chunxiao Li, Junjie Wang, Ping Jiang

**Affiliations:** Department of Radiation Oncology, Peking University Third Hospital, Beijing, China

**Keywords:** dosimetric impact, lymph node metastases, multileaf collimator systems, simultaneous integrated boost volumetric modulated arc therapy, stage IIIC cervical cancer

## Abstract

**Objective:**

Patients with FIGO stage IIIC cervical cancer frequently present with complex lymph node metastases (LNM), and proximity to multiple organs at risk (OARs) poses challenges for volumetric modulated arc therapy with simultaneous integrated boost (SIB-VMAT) planning. This study compared orthogonal dual-layer and parallel dual-layer multileaf collimator (MLC) systems, assessing dosimetric performance to inform clinical practice in complex nodal boost scenarios.

**Methods:**

This retrospective planning study included 45 patients with FIGO 2018 stage IIIC cervical cancer who underwent SIB-VMAT between February 2022 and March 2024. Patients were stratified according to LNM distribution into unilateral pelvic, bilateral pelvic, and para-aortic subgroups (n = 15 per subgroup). The planning target volume (PTV) received a prescribed dose of 45–50.4 Gy in 25–28 fractions, while metastatic lymph nodes (PGTVnd) received 54–63 Gy using the SIB technique. For each patient, SIB-VMAT plans were generated using an alpha orthogonal dual-layer MLC (α-MLC) system and a parallel dual-layer MLC (p-MLC) system under identical planning objectives and dose constraints. Dosimetric parameters of target volumes and OARs were analyzed to assess planning feasibility and system-dependent planning characteristics. Each system was evaluated within its clinically commissioned treatment planning environment to reflect real-world clinical practice.

**Results:**

Clinically acceptable target coverage was achieved with both MLC systems across all LNM subgroups. Particularly in patients with bilateral pelvic LNM, the α-MLC system demonstrated slightly improved dose gradient control for the PTV, reflected by a lower gradient index (p < 0.05). Other target-related dosimetric parameters for the PTV and PGTVnd, including D_max_, D_2_, D_98_, V_95_, V_110_, conformity index, and homogeneity index, were comparable between the two systems. Differences were observed in OAR sparing, with the α-MLC system consistently reducing doses to the small intestine, colon, rectum, bladder, femoral heads, cauda equina, and spinal cord across the overall cohort and within each LNM subgroup (p < 0.05).

**Conclusion:**

Both MLC systems achieved clinically acceptable target coverage across all LNM subgroups, while the α-MLC system demonstrated superior OAR sparing. These system-level differences may inform equipment selection and planning strategies in challenging nodal boost scenarios for advanced cervical cancer.

## 1 Introduction

Lymph node metastasis (LNM) is a critical prognostic factor in cervical cancer (1–3), with both the number and size of involved nodes inversely correlating with patient survival, highlighting the importance of effective regional nodal control (4–8). The 2018 revision of the International Federation of Gynecology and Obstetrics (FIGO) cervical cancer staging system incorporated LNM into the classification for the first time to standardize disease evaluation and guide treatment stratification, with pelvic lymph node metastasis classified as stage IIIC1 and para-aortic lymph node metastasis as stage IIIC2 (9). Patients with FIGO stage IIIC cervical cancer often present with multiple, widely distributed nodal metastases, and insufficient regional nodal control may result in residual disease and tumor recurrence, which can significantly reduce five-year survival and increase the risk of distant metastasis (10).

Radiotherapy remains a central component of definitive therapy for IIIC cervical cancer. In patients with locally advanced disease and LNM, achieving durable nodal control is essential for optimizing long-term outcomes. Studies have found that, on the basis of conventional fractionation doses (54–63 Gy), localized dose escalation to metastatic lymph nodes may improve local and regional control while reducing the risk of nodal recurrence (11–13). However, dose escalation also increases radiation exposure to normal tissues, including the bowel, bladder, and spinal cord, which may result in severe complications such as radiation enteritis and cystitis, substantially impairing patient quality of life. Therefore, achieving an optimal balance between dose escalation to the target to improve regional control and minimizing dose to adjacent organs at risk (OARs) while reducing treatment-related toxicities constitutes a key challenge in precision radiotherapy for locally advanced cervical cancer (14). In modern precision radiotherapy, volumetric modulated arc therapy (VMAT) is a widely adopted advanced intensity-modulated radiotherapy technique that delivers highly conformal three-dimensional dose distributions through continuous gantry rotation, dynamic multileaf collimator (MLC) motion, and real-time dose rate modulation. Compared with conventional static IMRT, VMAT enhances target dose homogeneity, reduces treatment time, and mitigates the impact of intra-fractional patient motion (15). Simultaneous integrated boost (SIB) enables differential dosing within a single treatment fraction, administering higher doses to high-risk targets, including metastatic lymph nodes, while maintaining standard prophylactic doses to elective regions without prolonging the overall treatment schedule (16). These advantages of SIB are highly complementary to the conformal dose delivery and treatment efficiency of VMAT, integrating into SIB-VMAT, which allows simultaneous escalation of nodal doses while protecting adjacent OARs (17). When targets comprise multiple, widely distributed lymph nodes in close proximity to critical OARs, the complexity of dose modulation increases substantially.

Under these conditions, the limited modulation capabilities of a radiotherapy system may result in suboptimal target coverage or excessive high-dose spill, potentially causing underdosing of lymph nodes or overdosing of adjacent OARs, thus compromising both treatment efficacy and patient safety. Consequently, plan quality increasingly depends on the integrated performance of the radiotherapy platform, encompassing both hardware and software components (18, 19). Among hardware-related factors, the design and dynamic motion characteristics of the multileaf collimator (MLC) play a critical role in beam shaping, dose gradient regulation, and sparing of normal tissues (20). Conventional parallel MLC shape beams through unidirectional leaf motion, whereas orthogonal MLC employ bidirectional leaf arrangement, theoretically enabling more flexible beam modulation, steeper dose gradients, and enhanced penumbra control for irregular targets. Simultaneously, treatment planning system (TPS) algorithms critically determine how these physical capabilities are translated into clinically deliverable dose distributions (21, 22). Accordingly, evaluating radiotherapy platforms as integrated systems is essential for understanding their performance in complex treatment scenarios, rather than attributing plan quality to isolated technical components.

Current studies comparing orthogonal and conventional parallel MLC in SIB-VMAT planning for cervical cancer patients with LNM remain limited. FIGO 2018 stage IIIC cervical cancer, with pelvic and/or para-aortic lymph node involvement, is commonly associated with targets that are extensive, irregularly distributed, and closely adjacent to critical organs, imposing stringent requirements on target conformity, dose gradients, and normal tissue sparing. Few studies have stratified patients according to the complexity of nodal distribution, which may directly influence the magnitude of dosimetric differences between MLC systems. It remains unclear whether orthogonal MLC can provide superior dosimetric performance relative to conventional parallel MLC in such complex clinical scenarios, and high-quality dosimetric evidence directly guiding clinical platform selection and plan optimization is still lacking. In this study, we systematically compared the dosimetric performance of orthogonal and parallel MLC in SIB-VMAT plans for patients with FIGO IIIC cervical cancer and complex nodal metastases, stratifying patients according to nodal distribution complexity. We aimed to delineate the dosimetric characteristics and relative advantages of each system, providing direct evidence to guide clinical platform selection and inform future strategies for dose escalation to metastatic lymph nodes.

## 2 Materials and methods

### 2.1 Clinical data

Forty-five consecutive patients with FIGO 2018 stage IIIC cervical cancer who received treatment at our radiation oncology department from February 2022 to March 2024 were retrospectively enrolled. The inclusion criteria consisted of histopathological confirmation of malignancy and radiological staging using CT and/or MRI. Stage IIIC1 was defined by radiological evidence of pelvic lymph node involvement, while stage IIIC2 was defined by para−aortic LNM. All patients underwent comprehensive pretreatment evaluations to rule out contraindications to radiotherapy. The cohort was divided into the unilateral pelvic LNM, bilateral pelvic LNM, and para−aortic LNM subgroups (n=15 each). This retrospective study was approved by the Institutional Ethics Committee (Approval No. (2023) 633-02), and written informed consent was obtained from all participants. The study was conducted in accordance with the Declaration of Helsinki and relevant national regulations.

### 2.2 Equipment and software

CT simulation was performed using a Philips Brilliance 16-row, 85-cm large-aperture CT scanner (Royal Philips, Netherlands). Treatment delivery was performed using the α-MLC system (VenusX) and p-MLC system (Halcyon 2.0) medical linear accelerators.

**Figure 1:**
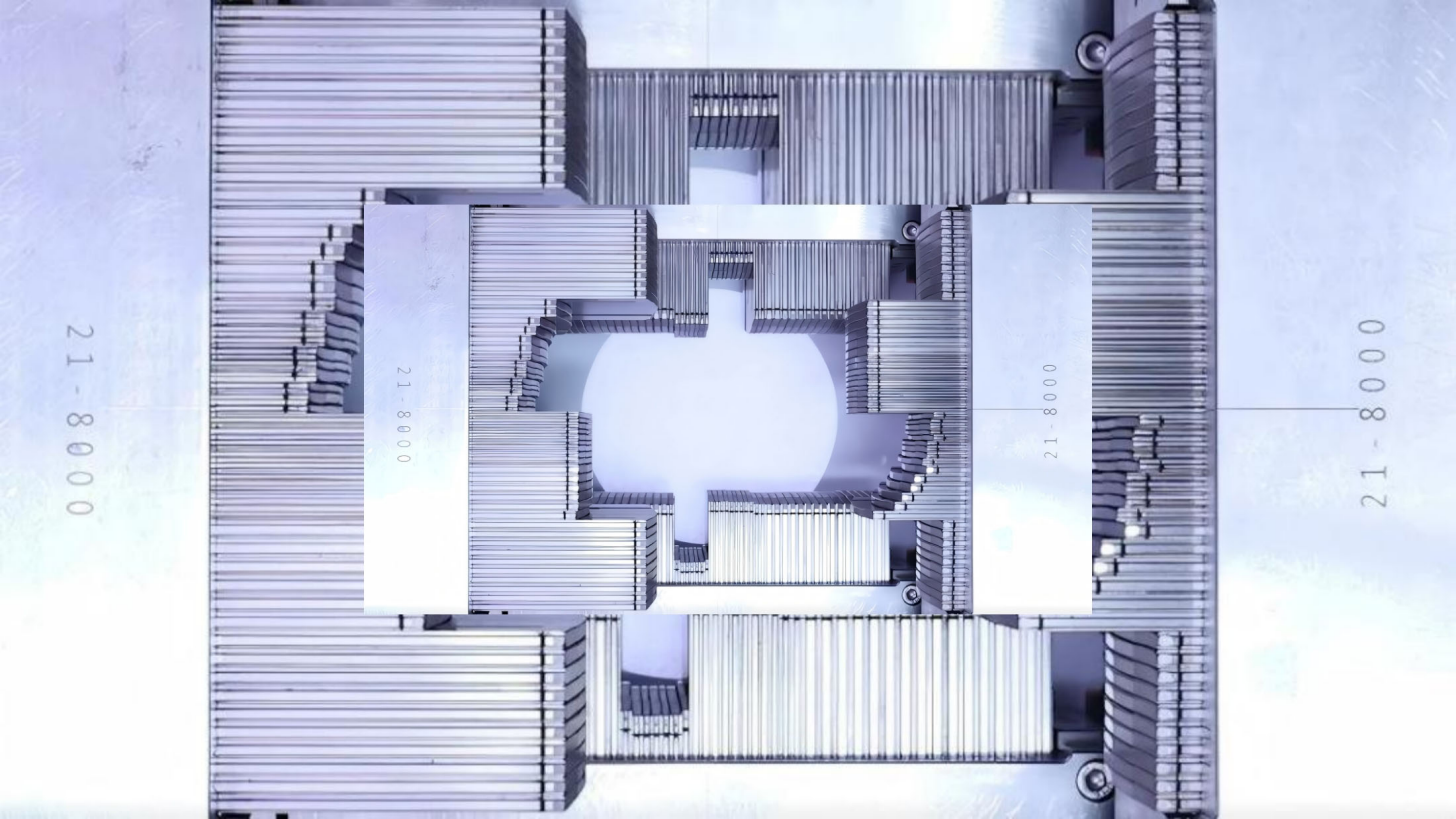
Schematic illustration of the orthogonal dual-layer multileaf collimator (α-MLC) configuration.

The α-MLC system (VenusX) linear accelerator (LinaTech TF Medical Science and Technology Co. Ltd., Beijing, China) is equipped with an orthogonal dual-layer MLC system (α-MLC), in which the upper and lower leaf layers move along mutually perpendicular directions (**Figure 1**) (23). Each MLC layer consists of two opposing leaf banks, with variable leaf widths designed to improve spatial resolution in the central treatment region. The orthogonal arrangement allows for effective field shaping in both in-plane and cross-plane directions, potentially improving conformity and dose gradient control. All leaves feature rounded ends to reduce interleaf leakage, and the system supports high-speed leaf motion suitable for VMAT. Treatment planning for the α-MLC system was performed using the TiGRT treatment planning system (TPS, version 2.0; LinaTech TF Medical Science and Technology Co. Ltd., Beijing, China), employing a Monte Carlo-based dose calculation algorithm with a calculation grid size of 3 mm.

**Figure 2:**
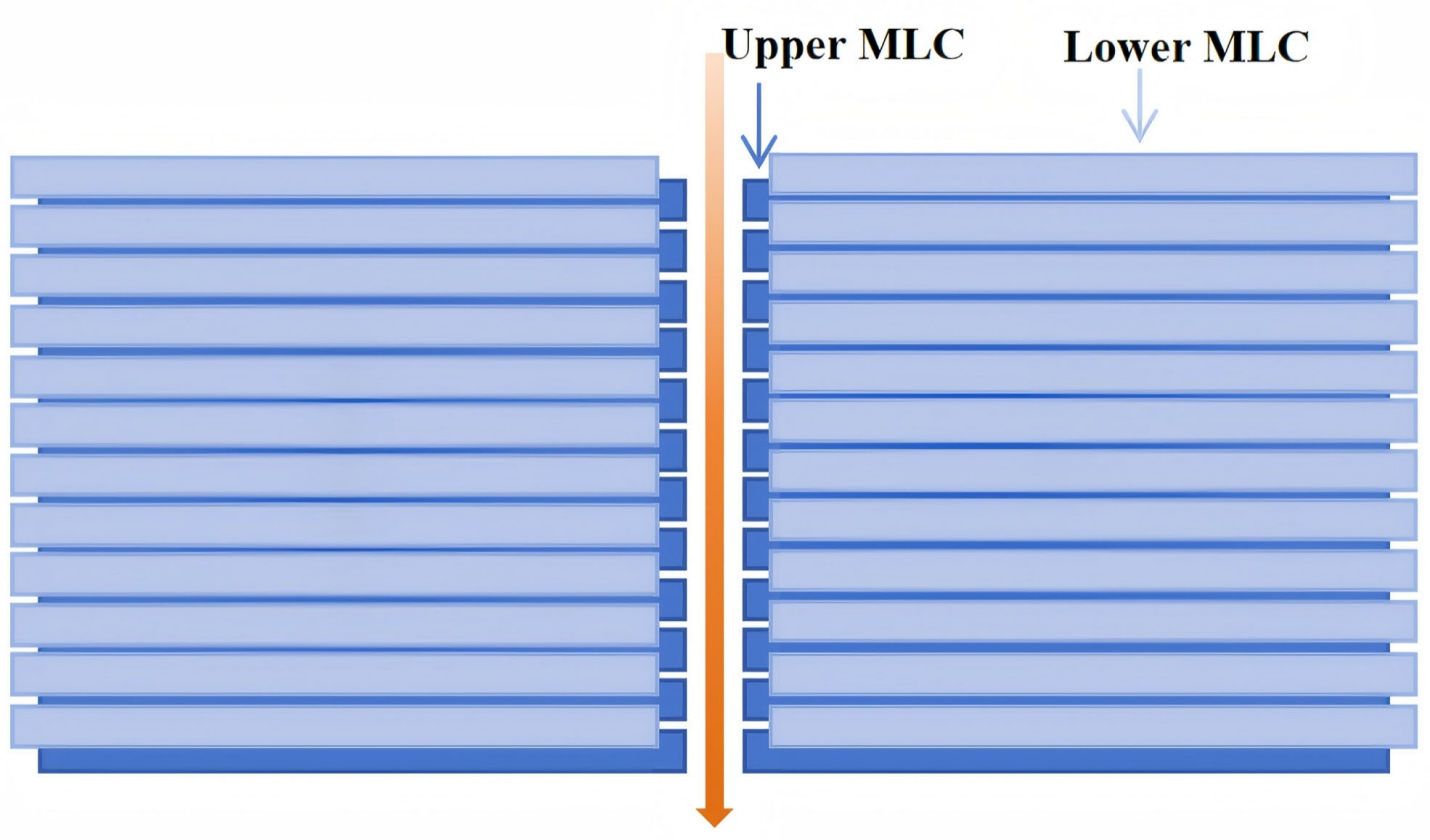
Schematic illustration of the parallel dual-layer multileaf collimator (p-MLC) configuration.

The p-MLC system (Halcyon 2.0) linear accelerator (Varian Medical Systems, Palo Alto, CA, USA) is equipped with a parallel dual-layer MLC system (p-MLC), in which the proximal and distal leaf layers are stacked along the beam direction and may move synchronously or independently (**Figure 2**) (24, 25). Each MLC layer consists of full-field leaf banks with uniform projected leaf width at the isocenter, and the dual-layer configuration improves transmission reduction and modulation efficiency compared with conventional single-layer designs. Treatment planning for the Halcyon 2.0 system was performed using the Eclipse TPS (version 15.0; Varian Medical Systems, Palo Alto, CA, USA) with the Analytical Anisotropic Algorithm (AAA) for dose calculation and a 2.5-mm isotropic dose grid. The key parameters of the two linear accelerators are summarized in **Table 1**.

**Table 1:** Technical specifications of varian halcyon 2.0 and LinaTech VenusX linear accelerators.

Category	Parameter	Varian halcyon 2.0	LinaTech VenusX
Accelerator Beam Characteristics	Photon Beam Type	6 MV FFF Photon Beam	6 MV FFF Photon Beam
	Secondary Collimator Design	No Movable Secondary Collimators	No Movable Secondary Collimators
	Source-To-Axis Distance (SAD)	100 cm	90 cm
	Maximum Gantry Rotation Speed	24°/s	7°/s
	Maximum Field Size	28×28 cm²	40×40 cm²
	Maximum Dose Rate	800 MU/min	1000 MU/min
MLC Structural Characteristics	MLC Configuration	Stacked and Staggered Dual-Layer MLC	Orthogonal Dual-Layer αMLC
	Leaf Motion Direction	Leaves Move Unidirectionally	Independent Motion Along Orthogonal X/Y Axes
	Total Number of Leaf Pairs	Distal Layer: 29 Pairs + Proximal Layer: 28 Pairs, Total: 57 Pairs	Upper Layer: 51 Pairs + Lower Layer: 51 Pairs, Total: 102 Pairs
	Leaf Width at Isocenter	Fixed 1 cm Per Leaf; Achieving 5 mm Effective Resolution Via Inter-Layer Staggered Design	Non-Uniform Distribution: Lower Layer 4.0/5.4/7.7 mm; Upper Layer 5.9/8.0/11.3 mm; Narrowest Width: Lower Layer 4.0 mm, Upper Layer 5.9 mm
	Field Penumbra	Leaf Edge: approx. 3.7–5.0 mm; Leaf Tip: approx. 4.1–6.9 mm	X-Direction: 6.2 mm; Y-Direction: 8.0 mm
	Maximum Leaf Speed	5 cm/s	Upper Layer: 7 cm/s; Lower Layer: 6 cm/s
MLC Dosimetric Characteristics	Leaf Transmission	Single Layer: Distal 0.41%, Proximal 0.40%; Dual Layer: 0.01%	Single Layer: 1.83–1.87%; Dual Layer: 0.03%
	Inter-Leaf Leakage	Single Layer: approx. 2–4%; Dual Layer: Negligible	Single Layer: 2.43%; Dual Layer: 0.17%

In this study, treatment plans for each patient were independently generated using two platform-level combinations: α-MLC with the Monte Carlo algorithm (TiGRT TPS) and p-MLC with the AAA algorithm (Eclipse TPS). Each MLC system is inherently paired and integrated with its dedicated TPS, dose calculation algorithm and optimization workflow, making this a platform-level system comparison rather than a pure MLC structural comparison; the independent contributions of each component to the study results cannot be separately isolated and quantified under this study design.

### 2.3 CT simulation, delineation of target volumes, and OARs

Contrast-enhanced CT simulation was performed for scan localization, covering the region from the superior border of the T10 vertebra to 5 cm below the ischial tuberosities, with a slice thickness of 5 mm. Target volume delineation strictly followed the guidelines of ICRU Report 83. At our institution, the gross tumor volume (GTV) for the primary cervical lesion was not delineated as a separate volume. The clinical target volume (CTV) included the uterine corpus, cervix, parametria, the proximal 3 cm of the vagina, and the pelvic lymphatic regions, with full parametrial coverage applied for tumors extending to the pelvic sidewall. Extended fields were delineated for patients with high−risk features or para−aortic LNM, with the superior border set at the level of the positive para−aortic node. Radiologically confirmed LNM in the pelvic or para−aortic regions were defined as the gross tumor volume of lymph nodes (GTVnd). Based on the experience of our institution’s Radiation Oncology Center, the planning target volume (PTV) was generated by applying anisotropic margins to the CTV. Margins of 6 to 8 mm were applied in the left to right and anterior to posterior directions, and 8 to 10 mm in the superior to inferior direction. The planning gross tumor volume of lymph nodes (PGTVnd) was generated by expanding the GTVnd by 5 mm. OARs included the small intestine, colon, rectum, bladder, spinal cord, cauda equina, femoral heads, bony pelvis, and kidneys.

### 2.4 Treatment planning design and standardization

#### 2.4.1 Prescription dose and organs at risk constraints

A standardized treatment planning protocol was established to define the dosimetric requirements for target volumes and OARs. The prescribed dose to the PTV was 45 to 50.4 Gy delivered in 25 to 28 fractions, with a fraction dose of 1.8 to 2.0 Gy administered five fractions per week. A SIB strategy was applied to the PGTVnd, escalating the total dose to 54 to 63 Gy within the same treatment course. Planning objectives were defined as follows: at least 95% of the PTV received at least 100% of the prescribed dose, and no more than 1% of the PTV received >110% of the prescribed dose, to ensure adequate target coverage and dose homogeneity. Dose constraints for OARs were defined as follows: V_45_ < 200 cm³ for the small intestine and colon; V_45_ < 50% and D_max_ < 57.5 Gy for the rectum and bladder; V_30_ < 15% and D_max_ < 55 Gy for the femoral heads. For extended-field irradiation, D_max_ to the spinal cord was limited to < 45 Gy, and D_mean_ to the kidneys was limited to < 10 Gy.

#### 2.4.2 Standardization of optimization parameters and beam arrangement

To ensure consistency in planning strategies between the two systems, optimization parameters were standardized. The optimization weight was set to 100 for the PTV and 50 for OARs. Identical dose constraints were applied to both systems to ensure comparability, with priority given to achieving adequate PTV coverage and adherence to prescribed dose limits, followed by further reduction of OAR doses within the allowable constraints. Beam arrangement was standardized using a dual-arc VMAT technique. The clockwise arc was delivered from 179° to 181°, and the counterclockwise arc from 181° to 179°. The collimator angle was fixed at 0°for both systems. The number of arcs, rotation ranges, rotation directions, and collimator settings were kept identical between the two systems to eliminate potential confounding effects of beam arrangement on dosimetric comparisons.

#### 2.4.3 Dose calculation algorithm and accelerator parameters

Both linear accelerators used 6 MV photon beams and underwent standardized calibration of beam flatness, symmetry, and absolute dose output prior to treatment. Dose calculation algorithms were validated using standard phantoms for both absolute and relative dose accuracy, ensuring consistency in baseline dosimetric performance. Dose calculation was performed using system specific configurations. The orthogonal multileaf collimator system used a Monte Carlo algorithm with a calculation grid of 3 × 3 × 3 mm³, whereas the parallel multileaf collimator system used an anisotropic analytical algorithm with a calculation grid of 2.5 × 2.5 × 2.5 mm³. These differences reflect the inherent characteristics of the two platforms in terms of dose calculation methods and accuracy.

#### 2.4.4 Quality control and reproducibility

To enhance reproducibility and minimize planner related bias, a rigorous quality control workflow was implemented. All SIB VMAT plans for each patient were generated by a single medical physicist with more than five years of clinical experience using linear accelerators equipped with parallel and orthogonal multileaf collimator systems. All plans met predefined requirements for target coverage and dose constraints for OARs prior to data export. All Digital Imaging and Communications in Medicine (DICOM) datasets, including computed tomography images, structure sets, dose distributions, and treatment plans, were anonymized by removing patient identifiers, resetting timestamps and operator logs, and randomizing file paths to reduce potential data-related bias. Subsequently, blinded plan reoptimization was performed on the VenusX platform by independent physicists. Each platform was optimized using its native treatment planning system with identical planning objectives to further minimize systematic bias and ensure the reliability and reproducibility of the results.

### 2.5 Plan verification

Patient-specific dosimetric quality assurance for both planning groups was conducted using the ArcCHECK cylindrical phantom. Prior to measurement, the linear accelerator’s static output was confirmed to be within ±1% to ensure the reliability of the dosimetric verification results. Dose discrepancies were evaluated using gamma analysis, applying a 3% dose difference and 2mm distance-to-agreement criterion to assess the accuracy of planar dose distributions. Although a gamma passing rate of ≥90% is commonly used clinically as an acceptance threshold, this study adopted a more stringent criterion of >95% to enhance verification rigor. Monitor units (MU) and beam-on times were recorded individually for each patient for subsequent analysis and quality assurance.

### 2.6 Evaluation parameters

The evaluation parameters for target volumes included D_max_ (Gy), D_2_ (Gy), D_98_ (Gy), V_95_ (%), V_110_ (%), conformity index (CI), homogeneity index (HI), and gradient index (GI) (26). Dosimetric metrics for OARs were as follows: small intestine and colon V_30_ (cc), V_40_ (cc), V_45_ (cc), and D_max_ (Gy); rectum and bladder V_30_ (%), V_40_ (%), V_45_ (%), and D_max_ (Gy); spinal cord and cauda equina D_max_ (Gy); bilateral femoral heads V_30_ (%) and D_max_ (Gy); pelvic bones V_10_ (%) and V_20_ (%); and bilateral kidneys D_mean_ (Gy). D_x_ is defined as the minimum dose received by X% of the volume of an organ or structure, and V_x_ (%) is defined as the percentage of the total volume of an organ or structure that receives a dose equal to or exceeding X Gy.

The conformity index was calculated according to Equation 1, where V_PTV_ denotes the volume of the planning target volume and V_Pres_ denotes the volume encompassed by the prescription isodose line (27). A CI value closer to 1 indicates better conformity of the treatment plan.

(1) $$\text{CI}=\frac{{\left(V_{PTV}\cap V_{pres}\right)}^{2}}{V_{PTV}\times V_{pres}}$$

The homogeneity index was calculated according to Equation 2, where D_2_, D_98_, and D_50_ represent the doses received by 2%, 98%, and 50% of the target volume, respectively, as derived from the dose–volume histogram (28). A lower HI indicates a more homogeneous dose distribution within the target volume.

(2) $$\text{HI}=\frac{D_{2}-D_{98}}{D_{50}}$$

The gradient index was calculated according to Equation 3, where V_1/2Pres_ represents the volume enclosed by the 50% prescription isodose line and V_Pres_ denotes the volume enclosed by the prescription isodose line (29). A lower GI indicates a steeper dose fall-off.

(3) $$\text{GI}=\frac{V_{1/2}\text{pres}}{V_{\text{pres}}}$$

All indices were calculated based on dose–volume histogram (DVH) data exported from the treatment planning system. Treatment delivery efficiency was evaluated by recording the monitor units and beam-on time for each plan.

### 2.7 Statistical analysis

Statistical analyses were conducted using SPSS Statistics (version 26.0; IBM Corp., Armonk, NY, USA). The normality of the differences between paired measurements was assessed using the Shapiro-Wilk test. Normally distributed data were presented as mean ± standard deviation (SD) and compared using the paired-sample t-test. Non-normally distributed data were presented as median (interquartile range, IQR) and compared using the Wilcoxon signed-rank test. All P-values were derived from two-sided tests, and a p-value < 0.05 was considered statistically significant.

## 3 Results

### 3.1 Comparison of key parameters

All 45 patients with FIGO stage IIIC cervical cancer successfully met the predefined planning objectives using both treatment systems. No statistically significant differences were observed between the α-MLC system plans and the p-MLC system plans with respect to target volume coverage, including D_max_, D_2_, D_98_, V_95_, V_110_, CI, HI, and GI for both the PTV and PGTVnd in the overall cohort (all p ≥ 0.05), indicating comparable target dose conformity and uniformity between the two systems. However, subgroup-specific differences in selected target-related parameters are detailed below.

In contrast, statistically significant differences were observed in multiple OARs dose parameters. Compared with the p-MLC system plans, the α-MLC system demonstrated consistently lower dose exposure to several critical OARs. Specifically, reductions were observed in dose–volume parameters of the small intestine (V_40_ and V_45_), colon (V_30_, V_40_, and V_45_), rectum (V_30_ and D_max_), bladder (V_30_, V_40_, V_45_, and D_max_), spinal cord (D_max_), cauda equina (D_max_), and bilateral femoral heads (V_30_) and left femoral head (D_max_), with all differences reaching statistical significance (p < 0.05).

Patient-specific quality assurance demonstrated high gamma passing rates for both systems under the 3%/2 mm criterion, with no statistically significant difference between the p-MLC system and α-MLC system plans (median 99.80% for both, p > 0.05). The α-MLC system plans required significantly higher MU than the p-MLC system plans, reflecting increased modulation complexity (1967.11 ± 327.35 vs 699.68 ± 161.93, p < 0.05). However, beam-on time was comparable between the two systems, with no statistically significant difference observed.

**Figure 3:**
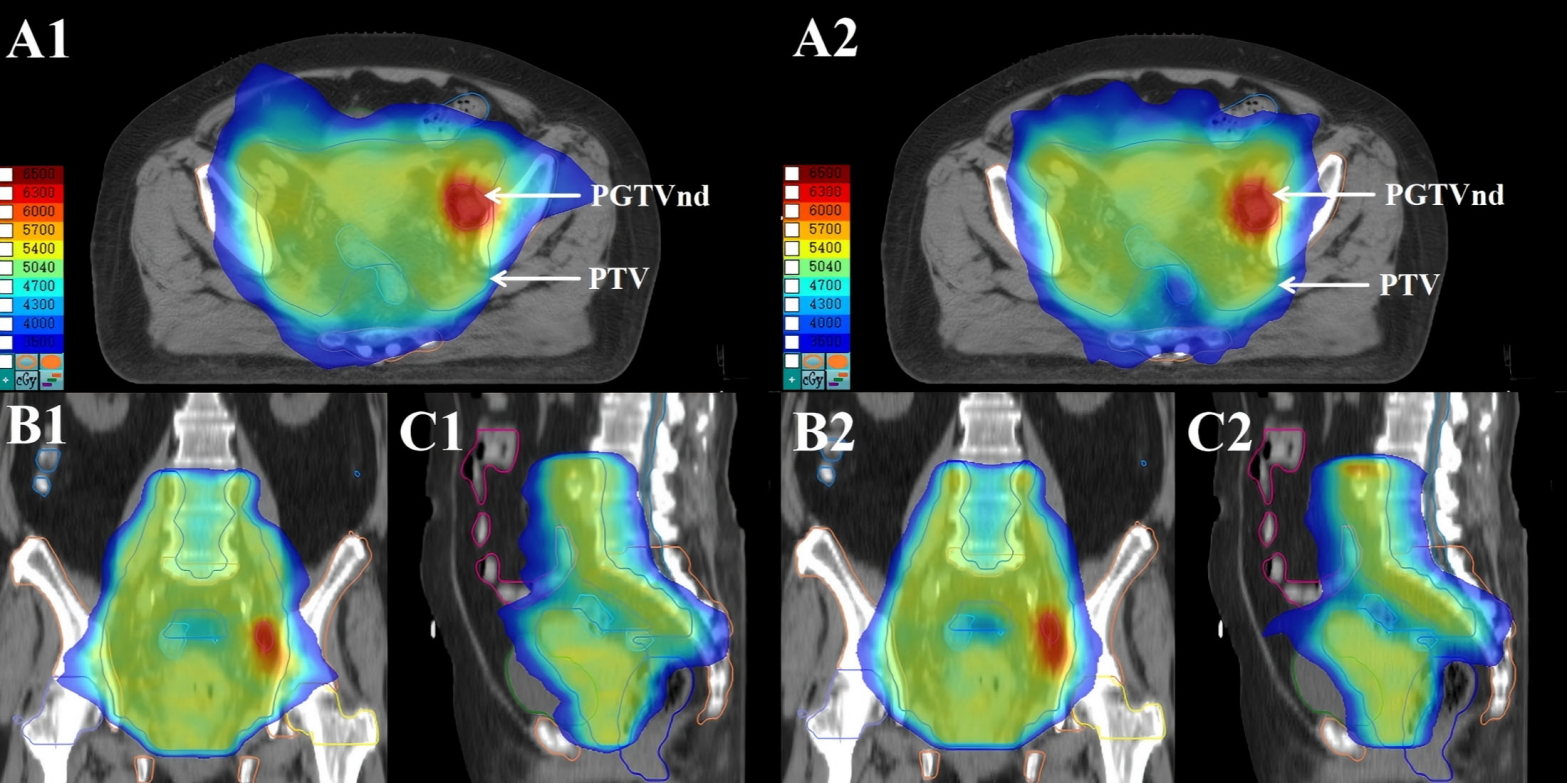
Dose distributions of p-MLC and α-MLC radiotherapy plans for a representative cervical cancer patient. (Axial, coronal, and sagittal views are shown in Panels **(A1,A2, B1,B2, C1,C2)**, respectively. Panels **(A1, B1, C1)** correspond to the p-MLC plan, whereas Panels **(A2, B2, C2)** correspond to the α-MLC plan.).

**Figure 4:**
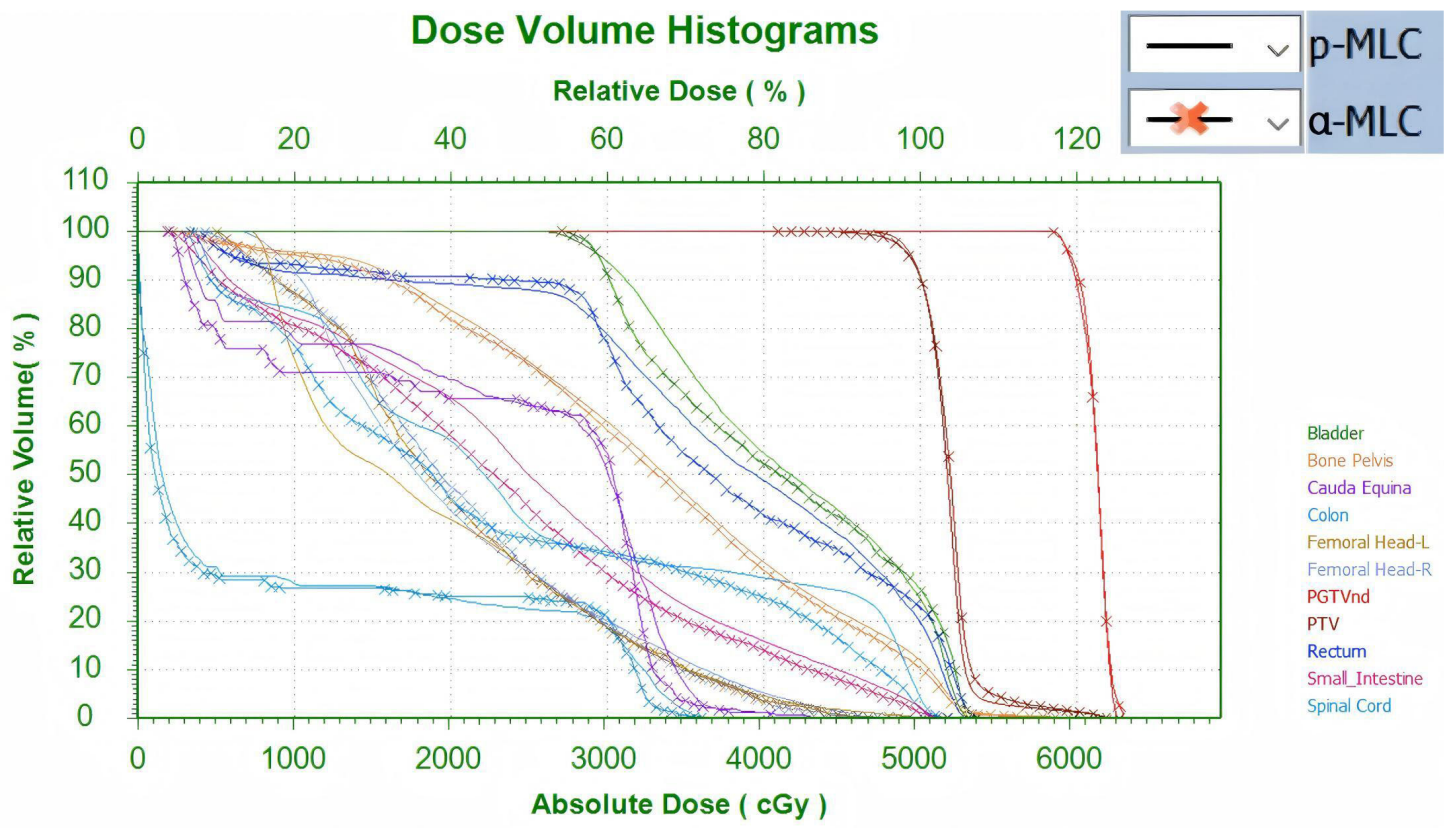
Comparative dose–volume histograms (DVHs) for p-MLC and α-MLC plans.

A summary of dosimetric parameters for target volumes, OARs, delivery accuracy, and treatment efficiency is presented in **Table 2**. Representative three-dimensional dose distributions and DVHs are shown in **Figures 3**, **4**, respectively.

**Table 2:** Comprehensive dosimetric comparison of target volumes, OARs, plan verification, and execution efficiency in a cohort of 45 patients.

ROI	Parameter	p-MLC system plan	α-MLC system plan	t/z	p
PTV	D_max_ (Gy)	63.36 (62.70, 64.11)	63.35 (62.80, 64.00)	1.15	0.25
	D_2_ (Gy)	60.63 (57.00, 61.99)	59.92 (57.20, 62.22)	0.98	0.33
	D_98_ (Gy)	49.04 (48.51, 49.34)	48.94 (48.31, 49.32)	0.62	0.54
	V_95_ (%)	99.44 (98.80, 99.63)	99.28 (98.74, 99.54)	1.27	0.20
	V_110_ (%)	6.62 (2.98, 16.53)	6.54 (3.52, 16.86)	0.10	0.92
	CI	0.86 ± 0.02	0.86 ± 0.03	0.81	0.42
	HI	0.23 (0.16, 0.25)	0.22 (0.16, 0.25)	0.79	0.43
	GI	3.87 ± 0.36	3.83 ± 0.34	1.43	0.16
PGTVnd	D_max_ (Gy)	63.34 (62.59, 64.06)	63.35 (62.68, 64.00)	0.93	0.35
	D_2_ (Gy)	62.91 (62.38, 63.50)	63.11 (62.61, 63.62)	1.52	0.13
	D_98_ (Gy)	59.10 (56.11, 59.48)	59.03 (56.29, 59.45)	0.07	0.95
	V_95_ (%)	99.94 (96.75, 100)	99.99 (96.59, 100)	0.36	0.72
	V_110_ (%)	0	0	-	-
Small Intestine	V_30_ (cc)	226.55 (157.71, 297.42)	207.05 (158.60, 300.98)	1.13	0.26
	V_40_ (cc)	112.33 (81.87, 169.64)	100.49 (70.55, 155.88)	3.38	0.001
	V_45_ (cc)	75.12 (47.62,117.69)	69.78 (45.28, 109.30)	3.70	0
	D_max_ (Gy)	53.26 (52.90, 54.06)	53.53 (52.97, 54.39)	1.26	0.21
Colon	V_30_ (cc)	98.20 (80.80, 129.14)	95.96 (83.16, 133.21)	2.41	0.02
	V_40_ (cc)	69.39 (55.80, 87.95)	67.53 (52.23, 80.77)	2.66	<0.01
	V_45_ (cc)	55.11 (41.59, 65.78)	51.18 (35.58, 62.62)	2.97	0.003
	D_max_ (Gy)	53.39 (52.95, 54.09)	53.56 (53.02, 54.36)	1.18	0.238
Rectum	V_30_ (%)	93.36 (83.68, 97.44)	91.01 (82.04, 98.12)	2.34	0.02
	V_40_ (%)	67.71 ± 15.62	67.79 ± 15.16	-0.12	0.91
	V_45_ (%)	54.02 ± 16.22	54.20 ± 16.57	-0.28	0.78
	D_max_ (Gy)	55.06 (54.16, 56.01)	54.70 (53.62, 55.33)	2.506	0.01
Bladder	V_30_ (%)	93.18 (83.51, 97.75)	91.17 (79.64, 96.71)	3.19	<0.01
	V_40_ (%)	65.81 ± 11.51	63.73 ± 12.33	3.04	<0.01
	V_45_ (%)	54.18 ± 10.87	52.14 ± 10.66	4.31	<0.0001
	D_max_ (Gy)	58.67 (55.85, 62.30)	58.61 (54.42, 62.01)	1.99	0.047
Spinal Cord	D_max_ (Gy)	38.07 (34.89, 40.69)	36.38 (33.04, 39.89)	4.20	0
Cauda Equina	D_max_ (Gy)	39.64 (36.51, 44.03)	37.35 (35.02, 41.31)	4.08	0
Femoral Head-L	V_30_ (%)	33.07 ± 12.83	27.95 ± 9.78	5.17	<0.0001
	D_max_ (Gy)	50.70 (46.63, 52.53)	47.73 (44.59, 50.56)	4.31	0
Femoral Head-R	V_30_ (%)	30.73 ± 12.84	25.09 ± 10.66	5.82	<0.0001
	D_max_ (Gy)	47.73 ± 4.02	45.77 ± 7.92	1.73	0.09
Bony Pelvis	V_10_ (%)	98.18 (96.51, 99.37)	98.31 (97.21, 99.25)	0.57	0.57
	V_20_ (%)	81.48 (77.85, 86.10)	82.13 (78.68, 86.28)	0.11	0.91
Kidney-L	D_mean_ (Gy)	3.38 (1.70, 12.39)	4.20 (1.35, 14.17)	0.18	0.86
Kidney-R	D_mean_ (Gy)	4.70 (1.94, 12.10)	4.56 (1.67, 14.11)	1.60	0.11
Plan verification	Gamma passing rate (3%/2 mm)	99.80 (99.65, 99.90)	99.80 (99.60, 99.80)	1.91	0.056
	Monitor Units (MU)	699.68 ± 161.93	1967.11 ± 327.35	-26.29	<0.0001
	Beam-on time (minutes)	1.58 (1.57, 2.10)	1.93 (1.70, 2.15)	1.27	0.20

ROI, Region of Interest.

### 3.2 Subgroup analysis of patients with unilateral pelvic LNM

In the subgroup of patients with unilateral pelvic LNM (n = 15), both treatment systems achieved equivalent target volume coverage. No statistically significant differences were observed in target-related parameters, including D_max_, D_2_, D_98_, V_95_, V_110_, CI, HI, and GI (all p ≥ 0.05). Compared with the p-MLC system plans, the α-MLC system plans demonstrated significantly lower dose exposure to multiple OARs. Notable reductions were observed in the small intestine (V_40_ and V_45_), colon (V_40_ and V_45_), rectum (D_max_), spinal cord (D_max_), cauda equina (D_max_), and bilateral femoral heads (V_30_) and left femoral head (D_max_) (p < 0.05 for all). Other evaluated OARs parameters did not differ significantly between the two systems. Detailed dosimetric results for this subgroup are summarized in **Table 3**.

**Table 3:** Dosimetric comparison of target volumes and OARs among unilateral pelvic LNM, bilateral pelvic LNM, and para-aortic LNM subgroups.

ROI	Parameter	Subgroup of 15 patients with unilateral pelvic LNM				Subgroup of 15 patients with bilateral pelvic LNM				Subgroup of 15 patients with para−aortic LNM			
		p-MLC system plan	α-MLC system plan	t/Z	p	p-MLC system plan	α-MLC system plan	t/Z	p	p-MLC system plan	α-MLC system plan	t/Z	p
PTV	D_max_ (Gy)	62.94 (61.99, 63.51)	63.07 (62.43, 63.72)	1.59	0.11	63.65 ± 1.85	63.61 ± 1.88	0.24	0.81	65.06 ± 2.52	65.10 ± 2.34	-0.32	0.75
	D_2_ (Gy)	58.05 (55.50, 61.90)	56.26(55.02, 62.22)	0.63	0.53	59.52 ± 2.97	59.78 ± 2.69	-1.74	0.10	61.98 (61.19, 63.33)	62.01 (60.87, 63.37)	0.17	0.87
	D_98_ (Gy)	48.59 ± 1.47	48.46 ± 1.40	1.41	0.18	48.92 ± 0.57	48.87 ± 0.48	0.90	0.39	48.96 (48.42, 49.26)	48.99 (48.29, 49.59)	1.34	0.18
	V_95_ (%)	99.33 ± 0.57	99.13 ± 0.55	1.90	0.08	99.24 ± 0.62	99.18 ± 0.49	0.82	0.43	99.08 ± 0.57	99.11 ± 0.52	-0.25	0.81
	V_110_ (%)	2.98 (2.11, 15.66)	3.53 (2.39, 14.27)	0.97	0.33	4.59 (3.4, 16.18)	5.82 (3.96, 8.27)	0.11	0.91	15.78 (7.65, 19.69)	16.84 (7.40, 20.20)	0.63	0.53
	CI	0.86 ± 0.03	0.85 ± 0.03	1.21	0.25	0.86 ± 0.02	0.86 ± 0.02	-0.30	0.77	0.85 ± 0.02	0.85 ± 0.03	0.67	0.51
	HI	0.17 (0.12, 0.24)	0.16 (0.13, 0.24)	1.00	0.32	0.20 ± 0.06	0.21 ± 0.05	-1.66	0.12	0.25 (0.23, 0.27 )	0.25 (0.22, 0.27)	1.48	0.14
	GI	3.83 ± 0.32	3.81 ± 0.32	0.44	0.67	3.98 ± 0.29	3.86 ± 0.25	2.45	0.03	3.80 ± 0.41	3.81 ± 0.40	-0.14	0.89
PGTVnd	D_max_ (Gy)	62.86 (61.99, 63.51)	63.07 (62.43, 63.72)	1.59	0.11	63.65 ± 1.85	63.57 ± 1.90	0.45	0.66	65.06 ± 2.52	65.10 ± 2.34	-0.32	0.75
	D_2_ (Gy)	62.56 (61.87, 62.93)	62.92 (62.40, 63.28)	1.93	0.053	63.40 ± 1.71	63.34 ± 1.68	0.30	0.77	64.47 ± 2.40	64.52 ± 2.35	-0.39	0.70
	D_98_ (Gy)	57.95 ± 2.55	57.99 ± 2.49	-0.40	0.70	59.20 (57.93, 59.44)	59.16 (57.14, 59.44)	0.28	0.78	59.09(54.16, 59.47)	59.03 (54.99, 59.35)	0.40	0.69
	V_95_ (%)	100 (97.14, 100)	100 (98.14, 100)	0.68	0.50	99.97 (,98.76, 100 )	100 (98.11, 100)	0	1	99.92 (95.53, 100)	99.94 (95.79, 100)	0.18	0.86
	V_110_ (%)	0	0	—	—	0	0	—	—	0	0	-	-
Small Intestine	V_30_ (cc)	222.63 ± 96.89	210.94 ± 95.35	2.01	0.06	234.92 (161.06, 328.40)	213.42 (168.49, 333.88)	0.34	0.73	227.40 (158.69, 306.04)	223.95 (159.77, 297.86 )	1.08	0.28
	V_40_ (cc)	124.89 ± 65.02	111.29 ± 65.45	3.62	<0.01	136.22 ± 62.83	122.41 ± 54.18	2.60	0.02	129.10 ± 64.01	127.46 ± 63.35	0.55	0.59
	V_45_ (cc)	86.40 ± 55.12	75.12 ± 52.22	4.67	<0.001	94.89 ± 49.28	86.29 ± 42.02	2.19	0.046	89.96 ± 50.31	88.06 ± 51.12	1.28	0.22
	D_max_ (Gy)	53.03 (52.81, 53.37)	53.27 (52.32, 53.63)	0.22	0.83	53.71 ± 0.81	53.81 ± 0.77	-1.30	0.22	53.59 (52.94, 54.21)	53.80 (53.25, 54.67)	1.48	0.14
Colon	V_30_ (cc)	107.11 ± 32.41	105.22 ± 30.30	0.85	0.41	106.58 (83.21, 134.28)	103.41 (87.36, 123.65)	1.31	0.19	112.95 ± 60.98	110.96 ± 60.51	1.32	0.21
	V_40_ (cc)	74.46 (61.30, 83.78)	72.49 (63.68, 88.61)	2.27	0.02	69.60 (55.98, 87.77)	66.83 (57.00, 79.36)	1.65	0.10	65.33 (39.62, 85.44)	67.39 (37.66, 90.60)	0.57	0.57
	V_45_ (cc)	61.40 (52.53, 76.33)	58.92 (49.53, 64.10)	2.22	0.03	55.61 (45.68, 63.01)	51.76 (40.29, 62.13)	1.76	0.08	47.44 (31.64, 58.99)	39.31 (30.46, 68.41)	1.25	0.21
	D_max_ (Gy)	53.03 ± 1.46	53.11 ± 1.29	-0.49	0.63	53.48 (53.08, 54.33)	53.78 (53.16, 54.34)	0.03	0.98	53.39 (52.67, 54.69)	54.00 (53.28, 55.50)	1.82	0.07
Rectum	V_30_ (%)	91.09 ± 5.56	90.48 ± 5.90	0.86	0.41	90.09 ± 9.24	87.35 ± 9.47	2.22	0.04	96.71 (83.22, 100)	96.12 (75.84, 100)	1.07	0.29
	V_40_ (%)	65.55 ± 13.22	66.29 ± 14.31	-0.66	0.52	67.27 ± 15.38	68.03 ± 14.28	-0.82	0.42	70.31 ± 17.11	69.04 ± 16.17	0.97	0.35
	V_45_ (%)	51.58 ± 14.88	51.64 ± 15.83	-0.28	0.96	54.43 ± 15.72	55.72 ± 16.05	-1.03	0.32	56.04 ± 17.12	55.25 ± 16.94	0.73	0.48
	D_max_ (Gy)	55.66 (54.02, 56.04)	54.79 (53.09, 55.25)	2.33	0.02	55.29 ± 1.96	54.75 ± 1.86	1.58	0.14	55.32 ± 1.50	55.04 ± 2.00	0.76	0.46
Bladder	V_30_ (%)	94.37(88.74, 99.00)	92.30 (79.25, 96.83)	1.43	0.15	89.46 (80.04, 96.33)	86.99 (80.04, 95.79)	2.17	0.03	90.39 ± 8.77	88.40 ± 9.31	1.44	0.17
	V_40_ (%)	63.51 (59.23, 72.28)	64.77 (59.82, 68.07)	0.45	0.65	64.61 ± 10.47	62.81 ± 11.74	1.34	0.20	65.82 ± 10.38	61.65 ± 10.14	3.87	<0.01
	V_45_ (%)	56.00 ± 13.86	55.06 ± 13.39	1.04	0.31	53.47 ± 9.62	51.68 ± 9.63	1.92	0.08	53.08 ± 7.55	49.68 ± 6.83	7.34	<0.0001
	D_max_ (Gy)	56.26 (54.61, 62.41)	56.13 (54.13, 61.87)	0.14	0.89	58.19 ± 3.45	57.51 ± 3.83	1.78	0.10	61.96 (57.05, 62.73)	61.00 (56.86, 62.28)	2.22	0.03
Spinal Cord	D_max_ (Gy)	34.54 ± 14.17	33.27 ± 13.44	2.48	0.03	33.91 ± 14.26	32.62 ± 13.85	2.17	0.048	38.07 (36.21, 39.45)	36.12 (34.33, 38.04)	3.11	<0.01
Cauda Equina	D_max_ (Gy)	40.71 ± 5.67	38.48 ± 5.98	3.15	<0.01	40.03 ± 6.81	37.39 ± 6.11	3.32	<0.01	38.20 ± 2.18	37.38 ± 2.66	2.04	0.06
Femoral Head-L	V_30_ (%)	33.51 ± 13.41	28.82 ± 11.00	2.15	0.049	33.80 ± 14.07	29.16 ± 10.21	2.97	0.01	31.89 ± 10.14	25.88 ± 6.99	4.29	<0.001
	D_max_ (Gy)	50.70 (44.21, 52.29)	47.97 (43.51, 50.63)	2.10	0.04	50.24 ± 3.59	48.78 ± 4.38	2.37	0.03	50.10 ± 3.36	47.66 ± 3.35	4.71	<0.001
Femoral Head-R	V_30_ (%)	29.70 ± 14.21	26.31 ± 12.93	2.79	0.01	32.67 ± 11.38	24.96 ± 7.66	4.28	<0.001	29.84 ± 12.10	24.01 ± 10.23	3.14	<0.01
	D_max_ (Gy)	47.21 ± 4.28	43.76 ± 12.30	1.03	0.32	48.44 ± 2.94	47.68 ± 3.31	1.39	0.19	47.54 ± 4.44	45.88 ± 3.74	2.60	0.02
Bony Pelvis	V_10_ (%)	97.70 (96.10, 98.92)	98.11 (97.19, 98.67)	0.35	0.73	98.40 (96.73, 99.55)	98.57 (97.15, 99.73)	0.66	0.51	98.93(96.49, 99.40)	98.57 (97.15, 99.42)	0.16	0.88
	V_20_ (%)	80.18 (77.12, 83.17)	80.41 (78.36 82.09)	0.41	0.68	81.34 ± 5.00	81.26 ± 5.37	0.10	0.93	84.77 (78.46, 87.76)	85.41 (81.13, 89.37)	0.41	0.68
Kidney-L	D_mean_ (Gy)	—	—	—	—	—	—	—	—	3.38 (1.70, 12.39)	4.20(1.35, 14.17)	0.18	0.86
Kidney-R	D_mean_ (Gy)	—	—	—	—	—	—	—	—	4.70 (1.94, 12.10)	4.56 (1.67, 14.11)	1.60	0.11

ROI, Region of Interest.

### 3.3 Subgroup analysis of patients with bilateral pelvic LNM

In patients with bilateral pelvic LNM (n = 15), target dose coverage was comparable between the two systems for most parameters. A statistically significant difference was observed only for the gradient index of the PTV, with the α-MLC system plans demonstrating a lower GI compared with the p-MLC system plans (3.86 ± 0.25 vs 3.98 ± 0.29, p = 0.03). No other target-related parameters showed significant differences (p ≥ 0.05). With respect to OAR sparing, the α-MLC system plans achieved significantly lower doses to several critical structures. Reductions were observed in small intestine V_40_ and V_45_, rectum V_30_, bladder V_30_, spinal cord D_max_, cauda equina D_max_, and bilateral femoral head V_30_ and left femoral head D_max_ compared with the p-MLC system plans (p < 0.05). The remaining OARs parameters did not differ significantly between the two systems. The detailed dosimetric results for this subgroup are presented in **Table 3**.

### 3.4 Subgroup analysis of patients with para-aortic LNM

For patients with para-aortic LNM (n = 15), no statistically significant differences were observed in target volume parameters between the two systems, including D_max_, D_2_, D_98_, V_95_, V_110_, CI, HI, and GI (all p ≥ 0.05). Compared with the p-MLC system plans, the α-MLC system plans demonstrated significantly lower dose exposure to selected OARs, including the bladder (V_40_, V_45_, and D_max_), spinal cord (D_max_), and bilateral femoral heads (V_30_ and D_max_) (p < 0.05). No statistically significant differences were observed in other evaluated OARs dose metrics. The detailed dosimetric results for this subgroup are presented in **Table 3**.

## 4 Discussion

Radiotherapy delivery in patients with FIGO stage IIIC cervical cancer with multiple LNM remains clinically challenging (10, 30). Extensive nodal involvement often spans large anatomical regions and abuts critical OARs such as the bowel and bladder, thereby increasing normal tissue irradiation and associated toxicity. To address these challenges, this study systematically compared the dosimetric performance of two linear accelerator platforms, α-MLC system and p-MLC system, in the generation of SIB-VMAT plans. The analysis included the entire cohort and three representative anatomical subtypes of LNM: unilateral pelvic, bilateral pelvic, and para-aortic involvement. Beyond fundamental assessments of target dose coverage, the study comprehensively evaluated OARs dose distributions, plan complexity, and treatment delivery consistency. The primary objective was to delineate the relative dosimetric strengths and limitations of these two systems in complex anatomical scenarios and to provide evidence to inform individualized radiotherapy equipment selection in clinical practice.

### 4.1 Target dose coverage equivalence and subgroup-specific differences

Accurate and reliable coverage of the target volume is a fundamental requirement for radiotherapy planning and directly influences the probability of achieving local tumor control (31). In the present study, both accelerator platforms achieved clinically acceptable target dose coverage across all subgroups, including the overall cohort, unilateral pelvic, bilateral pelvic, and para-aortic LNM, with key dosimetric parameters such as D_max_, D_2_, D_98_, V_95_ and V_110_ meeting predefined clinical objectives. With respect to dose distribution quality indices including the CI, HI, and GI, no statistically significant differences were observed between the systems in conformity or homogeneity across subgroups (all p ≥ 0.05) other than a significant difference in GI in the bilateral pelvic LNM subgroup. These results indicate that both systems demonstrate comparable target dose coverage, conformity, and uniformity. In the bilateral pelvic LNM subgroup, the α-MLC system showed a statistically significantly lower GI than the p-MLC system (3.86 ± 0.25 vs 3.98 ± 0.29; p < 0.05). The GI quantifies the steepness of dose falloff beyond the target boundary, with a lower GI corresponding to steeper dose attenuation and a potential reduction in the volume of normal tissues receiving low-dose irradiation. Because bilateral pelvic LNM inherently span both sides of the pelvis and are situated near critical structures such as the small bowel, colon, and bladder, a steeper dose gradient may theoretically limit incidental irradiation of adjacent normal tissues. This advantage may be attributed to multiple factors, including the enhanced modulation capability provided by the orthogonal dual layer leaf design of the α-MLC, the high accuracy of dose calculation achieved with the Monte Carlo algorithm, and optimized leaf motion speed and synchronization. The combined effects of these factors may contribute to improved edge dose control and reduced doses to OARs with the α-MLC system. Although this difference reached statistical significance, the absolute magnitude of GI reduction was modest, and no established clinical threshold currently links GI values to treatment-related toxicity or tumor control. Therefore, the clinical relevance of this finding should be interpreted with caution.

### 4.2 Dosimetric evidence for OARs sparing and considerations of clinical significance

Beyond ensuring adequate target dose coverage, sparing of OARs represents a critical component of radiotherapy plan quality, and sparing of OARs constitutes a critical component of plan quality that directly affects treatment safety and patient quality of life (32). Previous clinical studies have demonstrated clear associations between specific dose–volume parameters and radiation-induced toxicities. For example, increased bowel V_45_ has been linked to higher risks of acute gastrointestinal toxicity, while elevated bladder doses have been associated with both acute and late genitourinary complications (33–35). Radiation exposure to pelvic bone marrow has also been correlated with hematologic toxicity, and excessive dose to pelvic skeletal structures increases the risk of insufficiency fractures (36, 37).

In the present study, the α-MLC system achieved statistically lower dose metrics for several OARs. Specifically, in the overall cohort of 45 patients, the rectal D_max_ decreased from 55.06 (54.16, 56.01) Gy with the p-MLC system to 54.70 (53.62, 55.33) Gy with the α-MLC system (p=0.01), and the small intestine V_40_ decreased from 112.33 (81.87, 169.64) cc to 100.49 (70.55, 155.88) cc (p=0.001). Although these differences reached statistical significance, their absolute magnitude was generally modest, and not all evaluated OARs parameters consistently favored one system. Importantly, dosimetric improvements alone should not be interpreted as direct evidence of clinical benefit in the absence of toxicity or outcome data. Nonetheless, it is worth noting that in patients requiring extended-field irradiation or multiple nodal boosts, even modest reductions in dose across several OARs may have cumulative clinical relevance. From a practical standpoint, enhanced OARs sparing may offer greater planning flexibility when treating anatomically complex disease or when balancing dose escalation against normal tissue constraints.

### 4.3 Technical origins of dosimetric variations: contributions of hardware architecture and planning software attributes

The observed dosimetric differences should be interpreted within the context of a system-level comparison. In routine clinical practice, linear accelerators are commissioned and operated together with their manufacturer-specific treatment planning systems, and achievable plan quality reflects the combined performance of hardware design, dose calculation algorithms, and optimization strategies. Accordingly, this study evaluated the α-MLC system and p-MLC system platforms as integrated commercial systems under clinically realistic planning conditions, rather than isolating individual technical components.

#### 4.3.1 Differences in hardware design

Understanding the technical origins of dosimetric differences between the two systems may provide targeted theoretical insights to inform individualized radiotherapy equipment selection. Among the technical factors that influence dosimetric outcomes, the hardware design of the MLC is one of the important contributors to the observed differences in dosimetric performance (20, 38–40). The α-MLC system employs an orthogonal α-MLC configuration in which two independent leaf banks oriented in orthogonal directions enhance spatial modulation capability, enabling finer control of beam shaping and dose modulation (23). A key advantage of the dual-layer design is the synergistic shielding effect, which may reduce geometric penumbra and lower leaf-end transmission, both recognized determinants of sharper dose gradients at the interfaces between targets and adjacent OARs. Sharper dose gradients can theoretically reduce low-dose exposure to adjacent normal tissues and thus contribute to improved OAR sparing. Additionally, the combination of narrower central leaves and wider peripheral leaves in the α-MLC system design aims to maintain conformity while reducing mechanical complexity and improving the stability and precision of dose shaping. In contrast, the p-MLC system utilizes a p-MLC architecture, which inherently differs in leaf arrangement and motion characteristics (25). It is important to emphasize that this study did not perform an isolated component-level comparison of α-MLC versus p-MLC performance, but rather assessed the overall dosimetric behavior of two complete commercial systems. Therefore, although differences in MLC design are hypothesized to be an important hardware contributor to the dosimetric differences observed in this study, this association requires further validation through dedicated component-level comparative research.

#### 4.3.2 Software characteristics

At the software level, differences in dose calculation and optimization algorithms may have contributed to the observed dosimetric variations. The α-MLC system employs a Monte Carlo–based algorithm, which is widely recognized for its high accuracy, particularly in heterogeneous tissues (41, 42). The p-MLC system uses the AAA, which offers efficient computation and is clinically validated but relies on simplified physical modeling (43). vidence from prior studies suggests that Monte Carlo algorithms generally outperform analytic algorithms such as AAA in regions with substantial density variations, and advanced algorithms can further enhance dose accuracy and OAR sparing in complex anatomical scenarios (44). Within the pelvic region, where tissue composition is relatively homogeneous, algorithm-related differences may be less pronounced. Nonetheless, local heterogeneities, such as bowel gas interfaces, can still affect dose calculation, indicating that algorithmic differences likely contributed, at least in part, to the observed dosimetric discrepancies. Additional factors, including beam modeling, source-to-axis distance, and MLC representation within each planning system, may also influence dose outcomes. Dose calculation grid size represents another critical factor (45, 46). In this study, the Eclipse-based p-MLC platform employed a finer 2.5 mm grid, whereas the TiGRT-based α-MLC platform used a coarser 3 mm grid. Finer grids enable more accurate representation of dose gradients and local heterogeneity, whereas coarser grids may introduce smoothing effects that systematically affect spatially sensitive metrics, such as the gradient index, low-dose volumes, target conformity, and characterization of hot and cold spots (47). Although the overall trends observed are robust, absolute values of these metrics should be interpreted with caution. Collectively, these findings underscore the importance of evaluating radiotherapy platforms as integrated systems, particularly for anatomically complex cases.

### 4.4 Treatment delivery accuracy and clinical applicability

Reliable and reproducible treatment delivery is essential to realize dosimetric advantages. In this study of 45 patients, patient-specific quality assurance demonstrated high gamma passing rates under the 3%/2 mm criterion for both systems, with the p-MLC plans achieving a median of 99.80% (range 99.65–99.90) and the α-MLC plans 99.80% (range 99.60–99.80), with no statistically significant difference (p > 0.05). These results confirm strong agreement between calculated and delivered doses and demonstrate accurate SIB-VMAT delivery in clinically relevant settings. For the α-MLC system, which is equipped with an orthogonal dual-layer MLC, plans were more complex, with smaller and more numerous subfields, reducing beam utilization efficiency per MU and thereby leading to a significant increase in total MU (48). Across the cohort, the p-MLC plans had an average MU of 699.68 ± 161.93, whereas α-MLC plans had an average MU of 1967.11 ± 327.35, with a statistically significant difference between systems (p < 0.05). Median beam-on times were 1.58 minutes (range 1.57–2.10) for p-MLC and 1.93 minutes (range 1.70–2.15) for α-MLC, with no statistically significant difference (p > 0.05). Independent perpendicular motion of the two α-MLC layers enables finer spatial modulation, particularly advantageous for complex target geometries and multi-target irradiation. To achieve high-precision modulation, treatment plans incorporated additional control points, generating smaller and more numerous subfields and increasing leaf motion complexity, which reduced beam utilization efficiency per MU. Hardware kinematic constraints, including maximum leaf speed and interlayer interference, further contributed to MU escalation by potentially reducing dose rate or redistributing dose weights at certain control points. Despite the higher MU in α-MLC plans, the constant high dose rate of 1000 MU/min—substantially exceeding the variable dose rate of single-arc p-MLC plans (98–539 MU/min)—maintained beam-on times comparable between systems. Clinically, higher MU allows finer dose sculpting, improved OAR sparing, and steeper dose gradients at target edges, providing clear dosimetric advantages for complex targets. However, it may accelerate hardware wear, increase maintenance requirements, slightly elevate patient whole-body scatter dose, and complicate plan modulation and system operation. Therefore, implementation of the α-MLC should be accompanied by comprehensive quality assurance and real-time dose monitoring. For simple targets with regular geometry, these high-precision capabilities offer limited benefit and may reduce treatment efficiency. Maximum field size further differentiates the systems. The p-MLC is limited to 28 × 28 cm², whereas the α-MLC supports up to 40 × 40 cm². This advantage is particularly relevant for extended-field irradiation, as a larger treatment field reduces the need for field junctions and potentially decreases junction-related uncertainties, enhancing clinical versatility. These findings emphasize the need to individualize radiotherapy equipment selection based on target complexity, proximity to OARs, and treatment time considerations.

### 4.5 Study innovation and limitations

The innovations of this work include systematically evaluating the dosimetric differences between α-MLC and p-MLC designs across three lymph node distribution patterns (unilateral pelvic, bilateral pelvic, and para-aortic), covering the full spectrum of lymph node involvement in FIGO IIIC cervical cancer. In addition, rather than merely comparing MLC hardware, this study incorporates real-world TPS and dose calculation algorithms for platform-level assessment and quantitatively demonstrates the advantages of α-MLC in OARs protection and dose gradient control. These dosimetric advantages not only reduce exposure to critical organs but also provide additional planning margins and a reliable basis for potential escalation of lymph node doses, thereby offering clinicians greater flexibility in complex SIB-VMAT treatments. Several limitations should be acknowledged despite these evident benefits. This was a single-center, retrospective dosimetric study with a limited sample size, particularly within each LNM subgroup. Despite efforts to minimize planning bias through anonymization and blinded re-optimization, residual bias cannot be entirely excluded. Most importantly, the absence of clinical outcome and toxicity data precludes definitive conclusions regarding the clinical significance of the observed dosimetric differences.

## 5 Conclusions

Both the α-MLC and p-MLC systems were capable of generating clinically acceptable SIB-VMAT plans for patients with FIGO stage IIIC cervical cancer across diverse nodal distributions. The α-MLC provided certain dosimetric advantages in OAR sparing and modulation capability; however, these differences should be interpreted within the context of overall system performance rather than attributed to individual technical features. The higher monitor units associated with the α-MLC reflect increased modulation demand and may increase mechanical workload, yet beam-on time remained comparable, likely due to the high dose rate. The α-MLC may offer greater benefit in anatomically complex cases, whereas the p-MLC may be preferable for simpler targets where high-precision modulation provides limited additional benefit. Prospective multicenter studies incorporating clinical outcomes and toxicity endpoints are warranted to determine whether these dosimetric differences translate into clinically meaningful benefit. These findings support an individualized approach to treatment platform selection and VMAT planning strategies in complex nodal boost scenarios.

## Data Availability

The raw data supporting the conclusions of this article will be made available by the authors, without undue reservation.
